# THeragnostic utilities for neoplastic DisEases of the rectum by MRI guided radiotherapy (THUNDER 2) phase II trial: interim safety analysis

**DOI:** 10.1186/s13014-023-02353-x

**Published:** 2023-10-06

**Authors:** Giuditta Chiloiro, Angela Romano, Davide Cusumano, Luca Boldrini, Giulia Panza, Lorenzo Placidi, Elisa Meldolesi, Matteo Nardini, Guenda Meffe, Gianluca Nicolini, Claudio Votta, Luca Indovina, Maria Antonietta Gambacorta

**Affiliations:** 1 Fondazione Policlinico Universitario “Agostino Gemelli” IRCCS, Largo Agostino Gemelli 8, Rome, 00168 Italy; 2 Mater Olbia Hospital, Strada Statale Orientale Sarda 125, Olbia, 07026 Italy

**Keywords:** Magnetic resonance guided Radiation Therapy, Rectal cancer, Chemoradiotherapy, Early Regression Index, Radiomics, Dose escalation, Watch and wait

## Abstract

**Background:**

The THUNDER-2 phase II single institutional trial investigates the benefits of MRI-guided radiotherapy (MRIgRT) in treating locally advanced rectal cancer (LARC). This study focuses on evaluating the impact of escalating radiation therapy dose in non-responder patients using the Early Tumour Regression Index (ERI) for predicting complete response (CR). The trial’s primary endpoint is to increase the CR rate in non-responders by 10% and assess the feasibility of the delta radiomics-based MRIgRT predictive model. This interim analysis assesses the feasibility and safety of the proposed MRIgRT dose escalation strategy in terms of acute toxicity (gastrointestinal, genitourinary and haematological) and treatment adherence.

**Methods:**

Stage cT2-3, N0-2, or cT4 patients with anal sphincter involvement, N0-2a, M0, but without high-risk features were enrolled. MRIgRT treatment consisted of a standard dose of 55 Gy to the Gross Tumor Volume (GTV) and mesorectum, and 45 Gy to the mesorectum and drainage nodes in 25 fractions with concomitant chemotherapy. 0.35 T MRI was used for simulation imaging and daily alignment. ERI was calculated at the 10th fraction. Non-responders with an ERI above 13.1 received intensified dose escalation from the 11th fraction, resulting in a total dose of 60.1 Gy. Acute toxicity was assessed using the CTCAE v.5 scale.

**Results:**

From March 2021 to November 2022, 33 out of the total number of 63 patients to be enrolled (52.4%) were included, with one withdrawal unrelated to treatment. Sixteen patients (50%) underwent dose escalation. Treatment was well tolerated, with only one patient (3.1%) in the standard treatment group experiencing acute Grade 3 diarrhea, proctitis, and cystitis. No significant differences in toxicity were observed between the two groups (p = 0.5463).

**Conclusions:**

MRIgRT treatment with dose escalation up to 60.1 Gy is well tolerated in LARC patients predicted as non-responders by ERI, confirming the feasibility and safety of this approach. The THUNDER-2 trial’s primary and secondary endpoints will be fully analyzed when all planned patients will be enrolled.

**Supplementary Information:**

The online version contains supplementary material available at 10.1186/s13014-023-02353-x.

## Introduction

The achievement of pathological complete response (pCR) after neoadjuvant chemoradiotherapy (nCRT) in locally advanced rectal cancer (LARC) is associated with better disease local control (LC) and improvement in overall survival (OS) [1, 2]. Clinical complete response (cCR), defined as the absence of residual palpable masses on physical examination and on magnetic resonance imaging (MRI), can be used as a surrogate for treatment response and as guidance to address patients to conservative surgical approaches, such as local excision (LE) or watch and wait (W&W) [3, 4]. Obtaining a complete response (CR) is therefore a fundamental treatment milestone since it is a predictor of superior oncological outcomes.

It must be considered that the response to nCRT is highly variable in LARC, with pCR rates ranging from 11–42% [1, 5]. Dose escalation in radiotherapy (RT) is associated with increased tumor regression and improved pCR, and is currently considered a possible strategy to increase CR rates with acceptable acute toxicity rates [6, 7].

Different techniques and technologies can be applied to increase the dose on macroscopic disease, such as external beam RT, brachytherapy and contact therapy (CRX) in selected cases, but how to effectively delivery the dose boost still represents an open issue [8–10].

Although these techniques are generally characterised by favorable acute toxicity rates, it should also be considered that highly variable treatment-related toxicity rates impacting on quality of life (QoL) have been described, such as bowel dysfunction, urinary incontinence and sexual dysfunction [11, 12].

In addition to specific dosimetric issues, there is also great interest about which imaging technique represents the best solution to support the dose boost planning and determine treatment response. MRI has recently emerged as the main imaging modality for staging and treatment response assessment in rectal cancer [8].

In this context, MRI-guided radiotherapy (MRIgRT) appears to be the next logical step due to the advantages of being able to exploit daily MR imaging for innovative treatment planning and response monitoring throughout the treatment [13–15]. Furthermore, online adaptive (OA) RT solutions can be applied immediately prior to the delivery of the daily RT fraction, minimising the planning target volume (PTV) margins and potentially facilitating dose escalation and reducing the unnecessary irradiation of the surrounding organs at risk (OARs) [14].

Identifying possible factors that can predict response to nCRT, in order to be able to select patients for intensive therapy protocols, represents therefore an urgent and fascinating clinical challenge.

One of the most promising of such MR imaging-based biomarkers is the tumour early regression index (ERI) [16, 17]. This index takes into account the gross tumour volume (GTV) volumetric characteristics at the simulation phase and at the tenth treatment fraction, stratifying the patients into two subcategories, according to their probability to undergo pCR. The rationale of the THeragnostic Utilities for Neoplastic DisEases of the Rectum by MRI guided radiotherapy (THUNDER 2) trial, an ongoing prospective interventional phase 2 study, is to address patients considered as ERI “non-responders” to a RT dose escalation protocol up to 60.1 Gy on GTV + 0.3 cm isotropic margin using OA MRIgRT technique, starting from the eleventh treatment session. The primary aim is to increase the CR response by 10% in “non-responders” category, also investigating the feasibility of integrating delta radiomic-based predictive models with omics data to enhance predictive accuracy in MRIgRT. Secondary objectives include evaluating 3-year outcomes such as LC, metastasis-free survival (MFS), disease-free survival (DFS), OS, R0 resection rates (complete tumor removal), tumor regression grades (TRG) 1 and TRG 2, sphincter and organ preservation rates, as well as the preservation of rectal and sexual functions [18].

The main objective of this interim analysis is to report on feasibility and safety results in terms of acute toxicities.

## Methods and materials

### Study design and patient selection

This trial is a prospective interventional single-centre phase 2 trial, registered with ClinicalTrials.gov identifier NCT04815694. The trial has received the ethics approval from the ethics committee of Fondazione Policlinico Universitario “A. Gemelli”, IRCCS of Rome, Italy (ethics committee identifier code 3460). Before participating in the trial, each patient has to provide written informed consent.

All enrolled patients also undergo staging examinations including pelvic MRI, contrast-enhanced CT of the chest and abdomen or ^18^ F-FDG PET-TC according to clinical judgement, colonoscopy and histological examination. The patients are evaluated by a multidisciplinary team consisting of radiation oncologists, radiologists, surgeons, medical oncologists and pathologists.

To be eligible for inclusion in the trial, patients must meet the following criteria: age > 18 years, Eastern Cooperative Oncology Group (ECOG) 0–1, adequate haematological function, LARC cT2-3, N0-2 or cT4 for anal sphincter involvement N0-2a, M0, located between 0 and 15 cm above the anal verge.

However, patients with high-risk features such as mesorectal fascial involvement, extra-mesorectal nodal involvement, extra-mesorectal venous invasion (EMVI) and rectal mucinous adenocarcinoma histology were excluded from the study. Other exclusion criteria include previous pelvic RT, history of previous neoplasms (except for skin cancer and early cervical cancer), pregnancy and/or lactation, prior chemotherapy (CHT), severe comorbidities, or any condition that may affect adherence to the study protocol and follow-up [18].

Patients unable to undergo an MRI scan are excluded from the trial.

### Chemoradiotherapy treatment

All eligible patients were referred for nCRT by 0.35 T MRIgRT using MRIdian® Linac (ViewRay Inc, USA) system. The technical details of the treatment procedures have already been described in our earlier work on the study design [18]. After a medical screening for MR compatibility, the next step was the simulation phase. After acquiring and co-registering the 175-second 0.35 T MRI scan and the simulation CT scan in the same configuration and with the same immobilization systems, the contouring and planning phase was the next step. Contouring was performed according to the guidelines used at our Institution, considering CTV1 as the primary tumor and the corresponding mesorectum, and CTV2 as the total mesorectum and the pelvic drainage lymph nodes [19]. PTV1 and PTV2 were obtained by expanding CTV1 and CTV2 respectively by 0.5 cm [15]. Prescribed doses were 55 Gy to PTV1 and 45 Gy to PTV2 in 25 fractions of 2.2 Gy and 1.8 Gy per fraction, using a simultaneous integrated boost (SIB) 2 approach. Treatment plans were generated in inverse planning mode and normalised and validated according to the recommendations of ICRU 83 [20].

The GTV was contoured and independently checked by another experienced radiation oncologist in the field of rectal cancer on the MRI of the simulation and of the 10th therapy fraction for the calculation of the ERI index according to the following formula [16, 17]:

$$ERI= -ln\left[{\left(1-\left(\frac{{V}_{mid}}{{V}_{pre}}\right)\right)}^{{V}_{pre}}\right]$$

where Vpre is the GTV measured at the time of simulation and Vmid is the volume measured during therapy, at 10th fraction. GTV was defined as the primary rectal tumour seen on MRI scans taken during MRgRT treatment, including information obtained from diagnostic MRI scans. Patients with an ERI < 13 continued with the treatment planned at baseline. Patients with an ERI ≥ 13, classified as “non-responders”, underwent replanning. In the latter case, SIB3 treatment was replanned from the 11th session, aiming to increase the dose up to 60.1 Gy with daily fractionation of 2.54 Gy to PTV3, obtained by GTV + 0.3 cm isotropic margin.

In order to avoid any treatment-related toxicity and to exploit the potential of the MRIgRT technology, treatment in these cases was delivered using an OA strategy.

The dose was delivered using a cine-MRI gating protocol with a 5% region of interest (ROI) set within a 5 mm boundary from the CTV2 or 3 mm at the GTV in the case of dose escalation. This ensured the target volumes to be consistently positioned as planned during the procedure.

Five cycles of concurrent fluoropyrimidine-based CHT with 5-FU (225 mg/m^2^/day as continuous infusion) or oral capecitabine (1650 mg/m^2^/day as chronomodulation) were administered.

### Tumor response and toxicity evaluation

Early toxicity (within 6 months since nCRT end) and haematological profile were monitored weekly using the CTCAE version 5.0 scale [21] by routine blood tests and clinical examinations. Adverse effects were recorded in the patient’s clinical diary and in a dedicated electronic health record system. In case of ≥ G3 toxicity, both CHT and RT are discontinued according to protocol procedures, until symptoms subside.

Gastrointestinal, urogenital and haematological toxicities were assessed at approximately every 7 RT fractions. Renal function was also monitored for creatinine and azotemia, liver function by alanine transaminase (ALT), aspartate transaminase (AST), gamma-glutamyl transferase (GGT), direct and total bilirubin. Hemoglobin, leukocyte, neutrophil and platelet values were also recorded and inflammatory indices were calculated too [22].

According to the protocol schedule, patients were examined by rectal examination and clinically assessed for any toxicity (proctitis, diarrhoea, tenesmus, mucorrhoea, cystitis, fatigue, anaemia, leukopenia, thrombocytopenia) that occurred after CRT, approximately 45 days after completing nCRT.

The non-parametric Mann-Whitney U test for independent samples was used to compare toxicities between the two patient samples, “responders” and “non-responders”.

Six to eight weeks after the end of treatment, pelvic MRI and/or rectoscopy and total body CT were performed, depending on clinical judgement.

## Results

From March 2021 to November 2022, 33 patients were recruited. The median overall treatment time (OTT) was 35 days (range 32–53). Of these 33 patients, one patient withdrew from the study after 3 RT sessions due to cardiac problems unrelated to the ongoing CRT treatment, which required treatment to be interrupted, intensive cardiac care, and resumption of RT after six weeks. The patient was therefore excluded from the study and from this interim analysis.

Table 1 summarises the clinical characteristics of the 32 patients enrolled.

**Table 1:** Patient’s clinical characteristics

Patients characteristics	Number 32 (100%)
**Median Age years (range)**	67 (41–94)
**Gender** Male Female	18 (56.3) 14 (43.7)
**ECOG** 0 1	26 (81.3) 6 (18.7)
Smoking status Yes Not	8 (25) 24 (75)
**Comorbidities** Hypertension Type 2 diabetes Chronic obstructive pulmonary disease Benign prostatic hyperplasia Hypothyroidism Glaucoma Cardiological comorbidities (valvulopathy, atrial fibrillation, transient ischaemic attack, chronic ischaemic heart disease)	13 (40.6) 4 (12.5) 3 (9.4) 4 (12.5) 4 (12.5) 2 (6.3) 4 (12.5)
**Tumor location** High Middle Low	2 (6.3) 11 (34.4) 19 (59.3)
**cT stage** 2 3 4	4 (12.5) 26 (81.3) 2 (6.2)
**cN stage** 0 1 2	9 (28.1) 16 (50) 7 (21.93)
**Clinical stage** II III	9 (28.1) 23 (71.9)

Of the 32 patients treated, 16 (50%) were considered “responders” based on the calculation of the ERI index, while 16 (50%) were considered “non-responders” and were re-planned with a SIB3 treatment plan.

In all the latter cases, a daily OA approach was applied, from the eleventh treatment fraction onwards.

Out of a total of 800 delivered fractions, 209 (26.3%) were delivered with such OA modality.

Considering the first two weeks of treatment, overall 13/32 (40.6%) patients reported toxicity: 11 patients (34.4%) developed G1 toxicity (6 (18.8%) responders and 5 (25.6%) non-responders), while only 2 patients (6.3%) (1 of each category) developed G2 toxicity.

As for the overall toxicity distribution, 30/32 (93.8%) patients developed toxicity of any grade during CRT, of which 22/32 (68.8%) G1, 7/32 (21.9%) G2 and only one patient (3.2%) G3. These differences were not significant between the two groups using the Mann-Whitney U test (p = 0.54).

No G4-G5 toxicities were reported (Table 2).

**Table 2:** Distribution of toxicities for different grades in the two different study arms. *p value is referred to Mann-Whitney U test

32 Patients	Toxicity (CTCAE v 5.0) N (%)				
	G1 22 (68.8)	G2 7 (21.9)	G3 1 (3.2)	Total 30 (93.8)	*p value
16 boost	11 (34.4)	4 (12.5)	0	15 (46.9)	0.54
16 no boost	11 (34.4)	3 (9.4)	1 (3.2)	16 (50)	
Proctitis	7 (21.9)	1 (3.2)	1 (3.2)	9 (28.1)	
Diarrhoea	12 (37.5)	2 (6.3)	1 (3.2)	15 (46.9)	
Tenesmus	13 (40.7)	1 (3.2)		14 (43.9)	
Mucorrhoea	15 (46.9)	3 (9.4)		18 (56.3)	
Cystitis	8 (25)		1 (3.2)	9 (28.1)	
Fatigue	7 (21.9)	1 (3.2)		8 (25)	

Overall, the most common acute toxicities were diarrhoea 15/32 (46.9%), mucorrhoea 18/32 (56.3%) and tenesmus 14/32 (43.9%), followed by proctitis 9/32 (28.3%), cystitis 9/32 (28.3%) and fatigue 8/32 (25%). No haematological or other toxicity have been reported.

As shown in Table 2, which details types and distribution of the recorded toxicities, there were no significant differences between the two arms. Interestingly, the only case of G3 toxicity (diarrhoea and proctitis) occurred in the observation arm of the study.

**Fig. 1:**
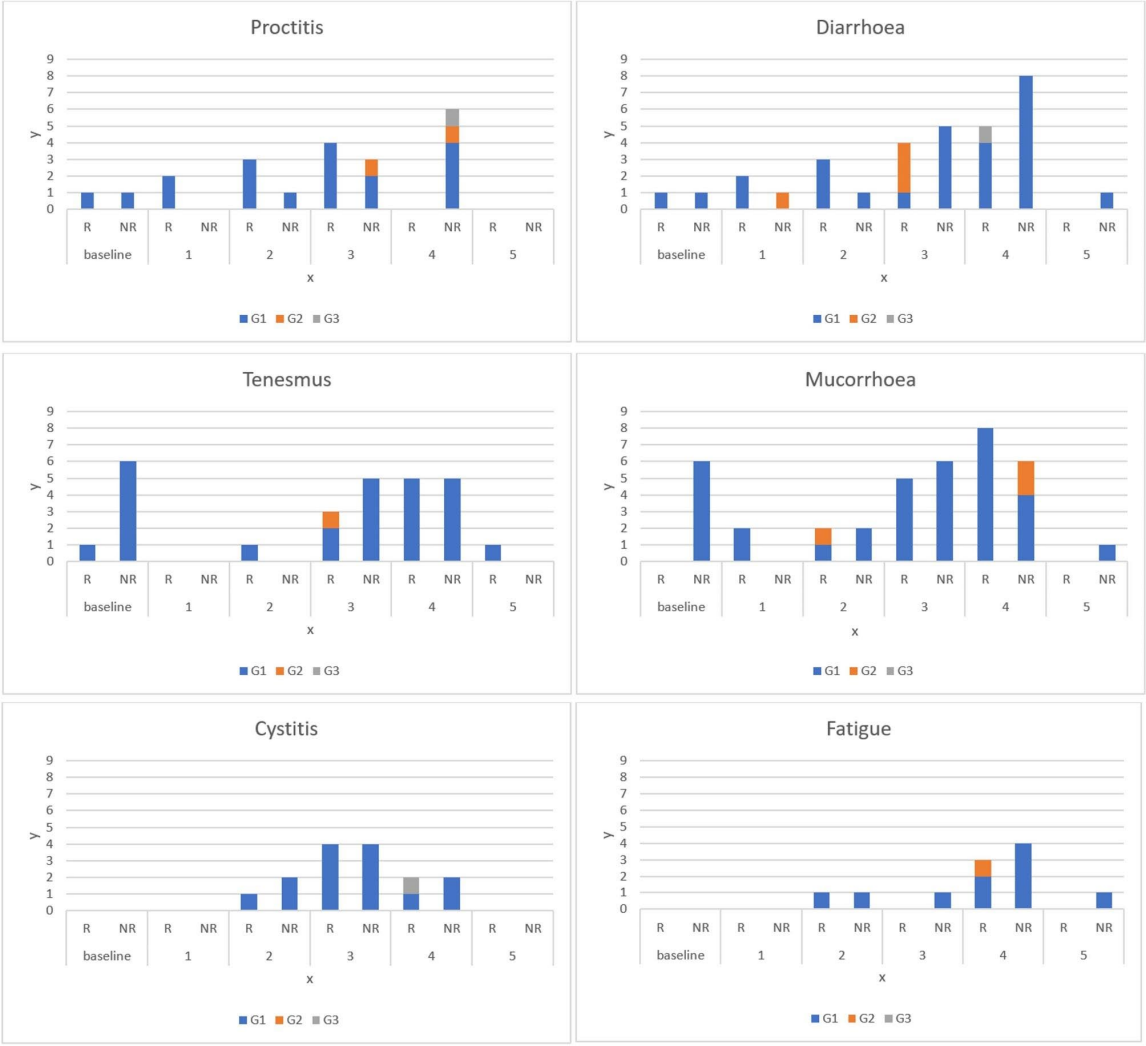
Trend of acute toxicities during chemoradiation treatment. On the x-axis, points 1 to 4 represent the clinical visits during treatment, every 7 RT fractions. Point 5 represents the clinical visit 45 days after the end of treatment. On the y-axis, the number of patients is displayed. R: responders; NR: non-responders

Figure 1 shows the trends of toxicities observed during RT. It should be noted that most of the toxicities occurred at the end of the third week (44.8%) and at the second week (27.6%) of treatment. Except for 4/29 (13.8%) patients who still had a mild G1 toxicity at the 45-day visit, all toxicities resolved earlier.

CRT treatment was discontinued in 5 (15.6%) patients for a median of 2 days (range 1–13) due to linac failure in 2 cases, G3 toxicity in 1 case, onset of fever which resolved spontaneously in 1 case, and inability of the patient to come to the hospital for treatment in 1 case; furthermore, CHT was discontinued in 2 other patients (overall 7 (21.9%)) due to the onset of hand-foot syndrome, with no differences between the two arms.

## Discussion

This study reports on the feasibility and acute toxicity results of the first cohort of patients enrolled in the THUNDER 2 study, treated with an OA dose escalation protocol during treatment according to an ERI-based predictive model on a hybrid MR-Linac device. The results obtained appear very promising considering that only 1 patient experienced G3 toxicity, notably belonging to the observational arm.

The most common toxicities of nCRT treatment for LARC have already been widely evaluated in several studies. They often seem to correlate with the synergistic effect of CHT and depend significantly on the volume of healthy tissue irradiated [23].

Common acute toxicities include fatigue, nausea, diarrhoea, proctitis, mucorrhoea and hematological toxicity. However, their severity and incidence can significantly vary depending on the specific patient population and selected treatment regimen. Furthermore, they are closely associated with the techniques used for radiation delivery.

A promising technology, able to introduce an innovative way of planning and reduce unnecessary irradiation of OARs is MRIgRT. It permits real-time imaging during treatment, leading to an easier and more reliable identification of the target volumes, better management of motion, and improved planning of adaptive treatments. The precise targeting facilitated by MRIgRT also allows for the delivery of higher radiation doses to the tumor, while efficaciously minimizing the exposure to healthy tissue. These dosimetric advantages contribute to improved clinical outcomes and a better compliance for patients undergoing nCRT in rectal cancer [14, 15].

For intermediate-risk LARC patients with a low potential for distant metastases, such as those enrolled in this trial, there is a need to explore strategies that can improve CR rates while minimising adverse effects and avoiding overtreatment. In this context, escalated dose RT appears to be a logical treatment option, as the potential side effects are primarily limited to nearby OARs [7].

These are the assumptions behind the design of THUNDER 2 trial, which aims to safely increase the CR rate and thus OS in LARC patients. This interim analysis shows a very favorable toxicity profile, with only one patient of the non interventional arm (3.2%) experiencing G3 toxicity, which quickly resolved with a short treatment discontinuation.

This appears to be a very promising result when compared with historical data.

Previously, in a case series of 22 patients treated with MRIgRT using the MRIdian® system (in its tri-60Co-60 version), 22.7% developed G3 toxicity. Although this result may be explained by the less conformed dose distribution of the Cobalt version of the MRIdian system®, it appears to be in accordance with those reported in the literature regarding G3 toxicity, which range from 10.3 to 40% [6, 24, 25].

However, there is one key aspect of the study that needs to be considered: 50% of patients were considered “non-responders” based on the application of the ERI-based predictive model and the dose was escalated up to 60.1 Gy on the GTV. Interestingly there were no significant differences in toxicities between the two arms (Table 2).

When looking at RT dose escalation studies in rectal cancer, toxicity rates are variable in the literature, with G3 toxicity rates varying from 10 to 42.6% [6]. A recent meta-analysis focused on dose escalation using volumetric modulated arc therapy (VMAT) and intensity-modulated radiotherapy (IMRT) technique in LARC reported a mean ≥ G3 toxicity of 11.06% (range 0–44%), with a mean G2 toxicity of 27.08% (range 6.8-49%). No correlation was found between dose regimen and toxicity [26].

Interestingly, also the use of endorectal brachytherapy for very selective tumour boosting is burdened by G3 toxicity rates up to 19.7% [7, 27].

When evaluating the existing evidence for boost delivery by MRIgRT, the data available in the literature is limited and heterogeneous.

Boeke et al. [28] analysed the results of 5 patients who underwent long-course nCRT with weekly fractions of response-adaptive boost at 3 Gy per fraction, with 3 cases of G3 toxicity during treatment that resolved at 6-month follow-up. Specifically, according to the PRO-CTCAE scale one patient reported acute G3 toxicity of the type diarrhoea, abdominal pain, dysuria, fatigue and nausea and the other two reported acute G3 urinary frequency. Liu et al. [29] enrolled 43 patients who were randomised to receive short-course RT with boost (25 Gy in 5 fractions plus 4 Gy to the GTV, followed by four cycles of CHT) or the long-course nCRT group (50 Gy in 25 fractions with concurrent CHT). The authors noted acute G3 or higher toxicities according to CTCAE scale 4.0 in 10/21 (47.6%) patients in the experimental group and 4/21 (19.0%) patients in the control group. The most common severe toxicities in the experimental group were proctitis, pain, dermatitis, leukopenia and diarrhoea in the control group.

There is every reason to continue the trial given the observed G3 toxicity rate of only 3.2%. It is probable that the highly advantageous toxicity profile observed in the present study can be attributed to the narrow safety margins (0.3 cm for the GTV and 0.5 cm for the larger volumes) made possible by the OA workflow and real-time image guidance provided by cine MRI for direct gating purposes.

The use of OA MRIgRT in nCRT for LARC has therefore showed superior dosimetric outcomes in comparison to conventional RT techniques, offering improved protection of nearby critical structures (i.e. small intestine, bladder, and femoral heads), resulting in a decrease in radiation-related toxicities [14].

Despite the confirmed feasibility and promising toxicity profiles, our study still presents some limitations. The number of patients is certainly limited to draw definitive conclusions, so we are waiting for the full sample of patients to be enrolled.

Clearly, acute toxicity is an assessment of feasibility and adherence, but it will be more interesting to know the late toxicities in terms of quality of life and long-term organ function, especially in patients who have reached cCR and undergone conservative approaches. In this context, it will also be of interest to know the rates of post-operative complications in the two arms of the trial for the total sample of patients.

## Conclusions

In conclusion, we successfully demonstrated the feasibility of a MRI-guided daily online adaptive radiotherapy dose escalation approach triggered by an image based predictive response model. This approach resulted in a very favourable rate of ≥ G3 adverse events. The completion of enrollment in this clinical trial should therefore proceed as scheduled.

### Electronic supplementary material

Below is the link to the electronic supplementary material.

Supplementary Material 1

## Data Availability

and supporting materials. The datasets analysed in the current study are available from the corresponding author on reasonable request.
