# Supplementary material for: THeragnostic utilities for neoplastic DisEases of the rectum by MRI guided radiotherapy (THUNDER 2) phase II trial: interim safety analysis
Source: Radiat Oncol. 2023 Oct 6;18:163. doi: 10.1186/s13014-023-02353-x (PMC10557246; doi:10.1186/s13014-023-02353-x)
Supplement: Supplementary file 1 — Supplementary Material 1 [file 13014_2023_2353_MOESM1_ESM.docx]

|  | WBC (White blood cells - Leukocytes (x10^9/L) | Hemoglobin (g/dL) | Platelet count  (x10^9/L) | Creatinine (mg/dL) | Urea, plasma (BUN)  (mg/dL) | AST (Aspartate aminotransferase)  (units/L) | ALT  (Alanine aminotransferase)  (units/L) | Gamma glutamyl transferase (GGT) | Bilirubin - tot  (mg/dL) | Bilirubin – Direct  (mg/dL) |
| --- | --- | --- | --- | --- | --- | --- | --- | --- | --- | --- |
| Baseline | 7,1 | 13,5 | 233,7 | 1,0 | 22,6 | 26,3 | 23,3 | 38,0 | 0,7 | 0,2 |
| 1 week | 6,2 | 13,4 | 222,6 | 1,0 | 22,4 | 17,8 | 14,3 | 29,1 | 0,8 | 0,2 |
| 2 week | 4,26 | 12,69 | 193,55 | 0,92 | 21,15 | 18,65 | 18,65 | 24,10 | 0,87 | 0,32 |
| 3 week | 4,13 | 12,54 | 171,07 | 0,98 | 21,07 | 20,21 | 15,06 | 22,82 | 24,44 | 0,35 |
| 4 week | 4,32 | 12,38 | 166,91 | 0,96 | 22,47 | 24,94 | 22,05 | 31,45 | 1,01 | 0,41 |
| 5 week | 4,10 | 12,29 | 173,75 | 0,91 | 17,60 | 21,50 | 24,00 | 28,22 | 1,12 | 0,45 |
| 45 days visit | 5,20 | 12,99 | 207,81 | 0,97 | 21,15 | 22,79 | 17,77 | 22,53 | 0,85 | 0,28 |

The table shows the average blood count and renal and hepatological function values of the patients enrolled at baseline, during CRT treatment and at the visit 45 days after the end of treatment.
